# Role of pancreatic lipase inhibition in obesity treatment: mechanisms and challenges towards current insights and future directions

**DOI:** 10.1038/s41366-025-01729-1

**Published:** 2025-02-27

**Authors:** Vetriselvan Subramaniyan, Yusoff Umul Hanim

**Affiliations:** 1 Pharmacology Unit, Jeffrey Cheah School of Medicine and Health Sciences, Monash University Malaysia, Jalan Lagoon Selatan, Bandar Sunway, 47500 Subang Jaya, Selangor Malaysia; 2 Division of Pharmacology, School of Medical and Life Sciences, Sunway University, Sunway, 47500 Malaysia

**Keywords:** Obesity, Metabolic syndrome, Fat metabolism

## Abstract

The worldwide health emergency of obesity is closely connected to how dietary fats are metabolized, whereas the process is significantly influenced by pancreatic lipase (PL), an enzyme critical for lipid hydrolysis into fatty acids. This narrative review employs a methodological approach utilizing literature searches of PubMed data up to March 2024. The search term criteria encompasses keywords related to the role, mechanism, challenges, and current and future treatments of pancreatic lipase in obesity with an overall references is 106. This paper offers a comprehensive explanation of the role of PL, underlining its significance in the digestive process and lipid imbalances that contribute to obesity and by extension, its impact on obesity development and progression. Additionally, it delves into the dual functionality of the pancreas, emphasizing its impact on metabolism and energy utilization which, when dysregulated, promotes obesity. A focal point of this review is the investigation into the efficacy, challenges, and adverse effects of current pancreatic lipase inhibitors, with orlistat being highlighted as a primary current drug delivery. By discussing advanced obesity treatments, including the exploration of novel anti-obesity medications that target specific biological pathways, this review underscores the complexity of obesity treatment and the necessity for a multifaceted approach. In conclusion, this paper emphasizing the importance of understanding the role of enzymes like pancreatic lipase mechanistic and adopting a multidisciplinary approach to treatment and side effects of current obesity drugs and explore new emerging therapeutic strategies for more effective obesity management.

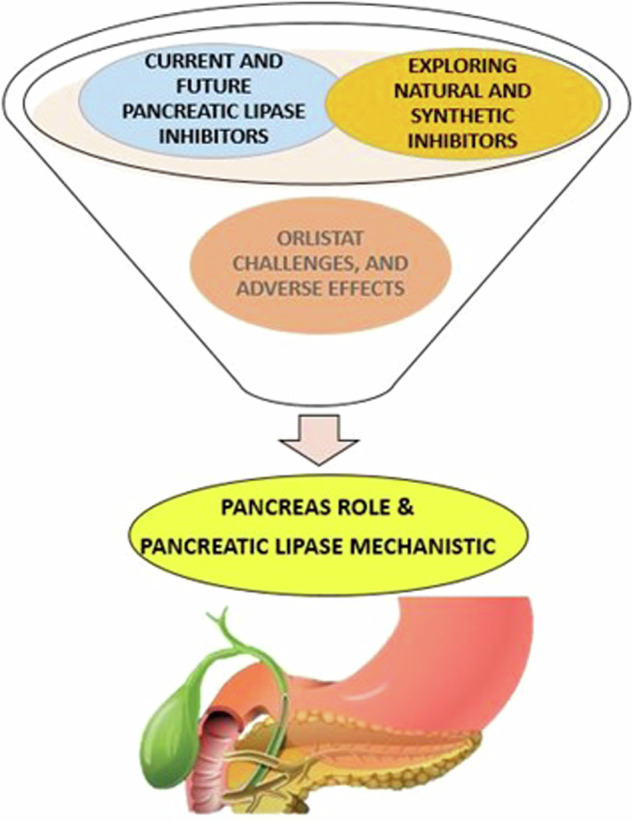

## Introduction

Obesity represents a global public health crisis, with its prevalence continually rising and contributing to a myriad of comorbid conditions, including type 2 diabetes [1], cardiovascular diseases [2], and certain forms of cancer [3], that impact millions of people around the world. It is a multifaceted issue marked by an excessive storage of fat, which leads to a heightened risk of many chronic illnesses. Traditional approaches to obesity management have primarily focused on lifestyle modifications [4], such as diet and exercise [5], and pharmacological interventions targeting general metabolic pathways. However, these strategies often fall short of providing long-term, sustainable weight loss, highlighting the urgent need for novel therapeutic approaches. In regard to the effective approach in combining pharmacological treatments with effective lifestyle modifications can indeed be challenging but holistic approaches offer a promising solution. Other than lifestyle balance, integrative medicine, such as complementary therapies like yoga and acupuncture manages to improve overall well-being. Collaboration in care model and patient education support offers an important lifestyle change, and providing ongoing support can enhance awareness and improve outcomes among communities.

Within this myriad strategy, our study offers a discussion on inhibiting pancreatic lipase (PL), which is a proven strategy for tackling obesity. This approach effectively interferes with the body's ability to store lipids, thereby aiding in weight loss [6]. Pancreatic lipase is a crucial enzyme in the digestive system that plays a role in the process of breaking down lipids into free fatty acids and monoglycerides [4]. This enzyme's activity is crucial for lipid absorption and metabolism, making it a compelling target for obesity intervention. The pancreas' unique role, combining both endocrine and exocrine functions, situates it as a key player in regulating metabolism [6]. When these roles are disrupted, it can greatly influence the onset and worsening of obesity, highlighting the importance of PL within the wider scope of metabolic well-being.

Recent studies have illuminated the effectiveness, obstacles, and possible adverse effects associated with existing PL inhibitors. Orlistat, the only FDA-approved pancreatic lipase (PL) inhibitor for long-term use, has been widely used as an anti-obesity drug in the U.S. and Europe for over a decade. Although effective, studies have reported that 91% of patients treated with orlistat experience gastrointestinal issues and other adverse effects [6, 7]. The evolution of new obesity drugs has been marked by significant challenges due to adverse effects, highlighting the need for future research to focus on safer, more targeted therapies with improved long-term outcomes. However, a large-scale study involving 33,625 patients found no significant link between orlistat use and an increased risk of colorectal cancer. Despite its benefits, the clinical application of orlistat is often hindered by its gastrointestinal side effects and modest weight loss results, prompting the search for alternative and adjunct therapies, including natural products and novel PL inhibitory peptides that may offer better tolerability and effectiveness in managing obesity [7–9].

Furthermore, the latest progress in peptide-based treatments [7] and the investigation of new anti-obesity drugs focusing on particular metabolic processes mark considerable progress in the evolution of advanced obesity therapies [9, 10]. These advancements highlight the intricate nature of excessive weight as a condition and the need for a comprehensive treatment strategy that includes innovative pharmacological solutions as a comprehensive management plan.

This manuscript aims to explore the significance of pancreatic lipase inhibition in obesity treatment, providing an overview of current insights and future directions. It delves into the mechanistic role of PL in obesity, examines the efficacy and limitations of existing inhibitors, and discusses the potential of emerging treatments. By highlighting the evolving landscape of obesity treatment and the critical role of ongoing research and development, this work underscores the potential of PL inhibition as a cornerstone of more effective and personalized obesity management strategies [10].

## Mechanistic insights into pancreas lipase

Among the vast array of biological mechanisms involved in the onset and advancement of obesity, the function of digestive enzymes, especially pancreatic lipase (PL), has attracted notable interest. Obesity is a multifaceted disease impacted by genetic, environmental, and lifestyle elements [5, 11]. Pancreatic lipase plays a key role in the digestive system, crucially breaking down dietary fats into fatty acids and monoglycerides that can be absorbed. This process is vital for the proper digestion and absorption of fats. When this process is disrupted, it can lead to imbalances in lipid levels and, ultimately, obesity or lipid absorption disorder [12, 13].

Recent advancements in molecular biology and biochemistry have shed light on the intricate mechanisms governing PL activity and its regulation within the gastrointestinal tract. Understanding these mechanistic insights is crucial for unraveling the complex interplay between dietary fat metabolism and the pathogenesis of obesity. This section seeks to provide a comprehensive overview of its role in energy homeostasis and its key role in managing and preventing obesity in the context of the significance of PL functions and related of the biochemical pathways.

### Overview of pancreas roles

**Fig. 1:**
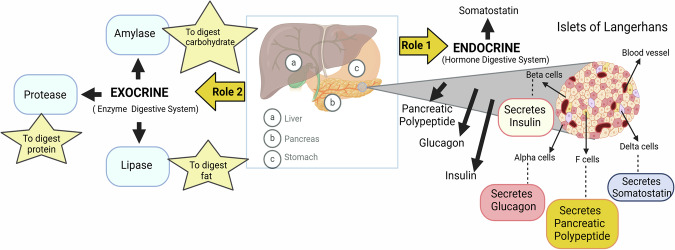
Role of pancreas as dual function-related endocrine and exocrine systems. The pancreas regulates blood glucose via insulin and glucagon, with dysfunction worsening diabetes and obesity.

Pancreas is a vital organ that serves key functions within both the digestive and endocrine systems. It is essential for the metabolism and breakdown of fats and proteins [14]. However, it is susceptible to a variety of diseases and disorders that can impact an individual's health severely. The pancreas has dual roles [6]; its exocrine function involves producing digestive enzymes like trypsin, chymotrypsin (proteases) for proteins, amylase for carbohydrates, and lipase for fats, along with pancreatic juices that aid in the digestion process in the duodenum (Fig. 1).

Its endocrine role is essential in regulating blood glucose levels, primarily through the secretion of insulin and glucagon. The pancreas extends horizontally across the abdomen, with its wider section situated on the right and narrowing towards the spleen. Various conditions can impact the pancreas, such as pancreatitis, pancreatic cancer, cysts, insufficiency, and fluid accumulation, each carrying significant health implications [15]. The pancreas plays a critical role in managing the body's metabolism and energy expenditure via its insulin production. However, disturbances in this function can lead to obesity by altering the manner in which the body stores and utilizes fats [16].

### Overview of lipid metabolism and enzymes

Lipids, which include fats, lipoids, and their derivatives, serve a variety of functions in the body. Triglycerides, or triacylglycerol, are the main form of stored fats found in adipose tissue and make up a significant portion of the body's weight in a healthy state [17]. Lipoids, such as phospholipids and glycolipids, are crucial for the structural integrity of tissues and play important roles in energy storage and consumption, particularly during times of high-energy demand. A small percentage of the body's total lipids, known as blood lipids, consists of phospholipids, triglycerides, cholesterol, free fatty acids, and small amounts of fat-soluble vitamins and steroid hormones [18]. These free fatty acids, mainly produced from the breakdown of triglycerides in adipose tissue, are vital for metabolic activities.

**Fig. 2:**
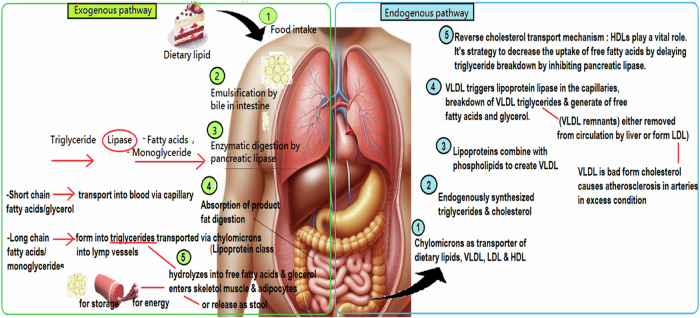
Lipid metabolism. Divided into two pathways, including exogenous and endogenous pathways.

The metabolism of lipids starts with the breakdown of ingested fats into smaller particles by bile, followed by the conversion into free fatty acids and monoglycerides by enzymes from the pancreas and small intestine [19] (Fig. 2). A portion of these fatty acids is further broken down into glycerol and fatty acids. Following their breakdown, substances such as glycerol and fatty acids with short-to-medium chains are directly taken up into the bloodstream through the walls of the small intestine. In contrast, monoglycerides and fatty acids with longer chains undergo reassembly into triglycerides within the cells of the small intestine [15]. These triglycerides are then combined with phospholipids, cholesterol, and proteins to create chylomicrons (transporter), which enter the circulatory system through the lymphatic route. The liver and pancreas play crucial roles in the various stages of lipid management, including digestion, absorption, production, and distribution within the body [20].

Lipases are key enzymes in the pancreas for this process, including pancreatic triglyceride lipase and its variants, which are essential for fat digestion, especially in newborns. Disruptions in the expression of these enzymes can affect fat digestion and increase the risk of pancreas and intestine diseases [21].

Pancreatic lipase plays a crucial role in the digestion process, specifically in converting dietary fats into monoacylglycerols and fatty acids within the duodenum. Its function is regulated by several factors to reduce side effects associated with medications and improve digestive efficiency [15]. This enzyme's activity is integral to the lipid metabolism pathway, which includes both the exogenous and endogenous pathways [14, 15]. The exogenous pathway involves the digestion and absorption starting from dietary fats, while the endogenous pathway manages the liver's production and distribution of fats within the body which involves the group of lipoprotein which is very low-density lipoprotein (VLDL) primarily responsible for transporting triglycerides (the most common type of fat in the body) from the liver to various tissues. After delivering triglycerides to tissues, VLDL is converted into low-density lipoprotein (LDL) referred to as bad cholesterol yet high-density lipoprotein (HDL) is good cholesterol [22].

### Unique role of pancreatic lipase in the duodenum: focuses on minimizing drug-related side effects

Pancreatic lipase (PL) is pivotal in the digestion process within the duodenum, specifically in breaking down dietary fats into absorbable molecules [14, 15, 22]. This process can be leveraged to address obesity by inhibiting PL activity, thus reducing fat absorption and caloric intake, which are essential for weight management. Despite its importance, the complete role of PL and other pancreatic enzymes in fat digestion is not fully understood, indicating a need for further research. Pancreatic enzyme replacement therapies that include PL have been beneficial for patients with pancreatic insufficiency, highlighting PL's therapeutic value.

The encoded PL involves the pancreatic lipase gene (PNLIP), which primarily acts in the duodenum by hydrolyzing triglycerides into monoacylglycerols and fatty acids [17]. Its activity can be enhanced by altering the phospholipid composition of fat droplets and is complemented by gastric lipase's partial hydrolysis of triglycerides. Pancreatic juice, which is rich in digestive enzymes including PL, plays a crucial role in digestion by neutralizing stomach acid and providing enzymes necessary for nutrient breakdown [19].

In the context of minimizing drug-related side effects, which is a major goal in medical treatment and drug development. Strategies such as gradual drug titration, understanding drug–target interactions for repurposing, system-wide approaches in drug development, optimized dosing, and improved drug delivery systems are all aimed at reducing these side effects [19].

The modulation of PL activity presents a unique opportunity to minimize side effects associated with drug therapy, especially for conditions related to dietary fat absorption. Previous studies explored the effects of apple polyphenol extract (APE) and procyanidin significantly reduced pancreatic lipase activity in vitro [23]. While other polyphenols in APE, such as catechins, chalcones, and phenol carboxylic acids, demonstrated minimal impact on lipase activity. The study found that the inhibitory power of pancreatic lipase is closely related to the degree of polymerization, with oligomeric procyanidin being a key contributor. Further investigation through a triglyceride tolerance test on both mice and humans revealed that consuming APE alongside triglycerides notably prevented the rise in plasma triglyceride levels in both subjects. This implies that oligomeric procyanidins within APE can reduce triglyceride absorption in the digestive system by targeting and inhibiting pancreatic lipase activity, making it a potential agent for managing triglyceride levels in the body potential in offering a new therapeutic approach [23].

Additionally, the new finding in current decades, i.e., the investigation of nutmeg’s capacity as a novel inhibitor of pancreatic lipase (PL) has been studied. Nutmeg, known for its culinary and medicinal uses, was examined for its effectiveness in blocking PL. Initial in vitro experiments revealed that nutmeg strongly inhibited PL activity by 66.24% [24]. Further investigations have identified a novel compound, tetrahydrofuran (THF), as a key player indicating the effective PL inhibitor. Collectively, these diverse research approaches strongly suggest that nutmeg, particularly through THF, holds potential as a natural inhibitor of pancreatic lipase, offering a promising strategy for obesity management by reducing the absorption of dietary fats [22, 24].

The choice of emulsifying agents and the role of micelles in PL activation are areas that could influence the development of interventions to control PL function effectively. Previous studies examined the impact of substituting traditional soybean phospholipid (SPL) emulsifiers with milk-derived polar lipid (MPL) emulsifiers in the diets of mice fed a high-fat regimen, focusing on obesity-related outcomes, such as adiposity and metabolic inflammation. The findings revealed that unlike SPLs, which contributed to an increase in the number of white adipose tissue (WAT) hypertrophy, inflammation, and liver steatosis, diets incorporating MPLs did not negatively affect adiposity or liver lipid concentrations [25]. Instead, MPLs were linked to beneficial effects on intestinal health, notably an increase in colonic goblet cells, suggesting an enhancement of the gut barrier. This improvement might stem from specific sphingomyelin (SM)-derived lipids in MPLs, which also correlated with reduced signs of macrophage infiltration in the WAT, indicating lower inflammation [25].

### The role of adipose tissue towards approaching to combat obesity

**Fig. 3:**
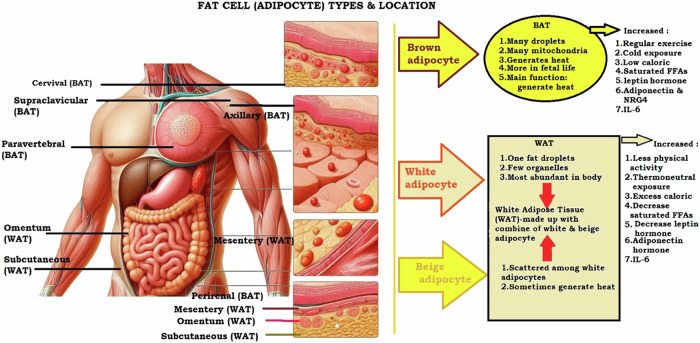
Role of adipocytes and location in the human body.

Research into adipose tissue identifies two main varieties: white adipose tissue (WAT), which is widely distributed across the body, and brown adipose tissue (BAT), which is found in particular locations like the neck, shoulder area, and around certain internal organs [26, 27], as illustrated in Fig. 3. White adipose tissue is primarily found beneath the skin in the abdominal, gluteal-femoral, and visceral regions, serving as energy storage, mechanical cushioning, insulation, and protection against infections [28]. Conversely, BAT is characterized by its ability to burn energy and produce heat, whereas it enriches mitochondria content and the action of uncoupling protein 1 (UCP-1), which helps in heat generation from fats. Besides its energy storage function, WAT is implicated in obesity due to its secretion of substances that regulate metabolism, inflammation, vascular function, and reproductive processes, including hormones like leptin and adiponectin, which influence body weight, and others that play roles in local inflammation and vascular health [28].

White adipose tissue (WAT) is vital in maintaining energy balance within the body, serving both as a repository for energy storage and as an endocrine entity that releases hormones like adiponectin and leptin. These hormones play crucial roles in managing metabolic functions, such as the oxidation of fatty acids, and are deeply involved in the pathophysiology of obesity [29, 30].

White adipose tissue (WAT) tends to proliferate under conditions of reduced physical activity, exposure to thermoneutral environments, consumption of calorie-rich diets, decreased levels of saturated free fatty acids, imbalance of leptin and adiponectin hormone levels, and increased interleukin-6 (IL-6). On the contrary, brown adipose tissue (BAT) expands with regular exercise, cold exposure, lower calorie diets, elevated free fatty acids (FFAs), increased levels of both leptin and adiponectin hormones, and a rise in IL-6. Adipocyte mainly summarizes the conditions that influence the accumulation or reduction of white and brown adipose tissues, highlighting the impact of lifestyle, environmental factors, and biochemical signals on body fat composition [29].

Adiponectin, part of the adipokines hormone released by fat cells, plays a crucial role in improving insulin sensitivity and managing blood sugar and lipid metabolism [29]. Unlike most fat-derived hormones, adiponectin levels tend to decrease with an increase in body fat percentage in adults, linking lower adiponectin concentrations with obesity [27, 29]. This hormone is instrumental in promoting the breakdown of fatty acids, which has significant implications for addressing obesity. It enhances the burning of fats in muscle and liver tissues by activating the adenosine monophosphate-activated protein kinase (AMPK). This essential enzyme regulates cellular energy balance by promoting energy-producing activities such as glucose absorption and lipid catabolism. The stimulation of AMPK in muscle and liver leads to more efficient fat breakdown, preventing fat buildup in these organs and thus contributing to obesity prevention. Furthermore, adiponectin supports the absorption of glucose by cells, which in turn lowers insulin production by the pancreas and decreases fat storage. This enhancement of insulin sensitivity guarantees a more efficient transformation of glucose and fats into energy, thus reducing their storage as body fat [28, 30].

Leptin, also secreted by white adipose tissue (WAT), functions as a hunger-suppressing signal that helps regulate energy equilibrium by curbing appetite, leading to decreased food consumption and increased energy utilization. Its involvement in lipid metabolism and the management of obesity extends to controlling energy expenditure [31]. High levels of leptin, which are common in individuals with obesity due to leptin resistance, fail to properly signal satiety, leading to continued food intake and reduced energy expenditure [32]. Although leptin primarily regulates appetite and satiety, it also has effects on glucose metabolism and fatty acid oxidation through its impact on insulin sensitivity. However, in the context of obesity, the effectiveness of leptin in promoting fatty acid oxidation is diminished due to leptin resistance. In the context of obesity, impaired secretion of adiponectin and leptin leads to decreased breakdown of fatty acids and heightened fat storage. Diminished adiponectin levels result in less effective fatty acid oxidation, causing fat to accumulate in tissues and potentially leading to insulin resistance. At the same time, resistance to leptin during obesity impairs the regulation of hunger and energy use, further contributing to fat accumulation [30–32].

The relationship between adiponectin levels and obesity is complex, with some individuals with obesity showing high adiponectin levels without associated metabolic problems, suggesting a nuanced interaction between adiponectin and obesity. This complexity is further heightened by differences in adiponectin production between visceral and subcutaneous fat, notably with obesity-associated declines in adiponectin from subcutaneous sources [33]. These intricate connections between hormones produced by adipose tissue and obesity underscore crucial mechanisms by which obesity fosters inflammation, insulin resistance, and metabolic conditions like type 2 diabetes. Understanding these processes offers opportunities for targeted treatments that focus on enhancing fat breakdown and metabolic health by modulating the effects of adiponectin and leptin [28, 29, 31].

## Current PL inhibitors and their efficacy

The rising global incidence of obesity is an urgent public health issue linked to numerous metabolic diseases, including type 2 diabetes, heart disease, and some cancers. The metabolism of dietary fats is key to the onset and advancement of obesity. Recent research efforts have concentrated on the development and assessment of inhibitors of pancreatic lipase as a strategy for addressing obesity, especially when linked to diets rich in fats [26]. These inhibitors work by limiting the digestion and absorption of fats, leading to reduced calorie consumption and supporting weight reduction [28]. Pancreatic lipase inhibitors, a category of medications aimed at obesity treatment, inhibit the pancreatic lipase enzyme. This enzyme's role is to break down dietary triglycerides in the intestines. By blocking this enzyme, triglycerides are not converted into free fatty acids that can be absorbed, resulting in their excretion without digestion. This process helps reduce the number of calories absorbed from fat, aiding in weight loss. Orlistat, one of the most well-known PL inhibitors, offers a case study on the effectiveness, challenges, and side effects associated with this therapeutic approach [24]. In this section, the article reviews the current landscape of pancreas lipase inhibitors, including synthetic drugs and natural compounds, their mechanisms of action, clinical efficacy, and safety profiles. Several FDA-approval drugs will be discussed, including liraglutide, semaglutide, tirzepatide, orlistat, and combinations like phentermine/topiramate and bupropion/naltrexone, noting their effectiveness in obesity management [34, 35] (Table 1).

**Table 1:** Adverse effects associated with current obesity treatment drug delivery information.

Name (FDA approval)	Used term	Principle	Delivery mode	Side effect
Phentermine (Adipex, Lomaira) [93]	Short-term (3 months)	Reduces hunger cravings	30 mg/day (Capsule/Tablet)	Blood pressure disorder
Diethylpropion (Tenuate) [94]	Short term	Adjustment of norepinephrine activity	50 mg/2 times/day	Sleep disorder, xerostomia symptom
Phentermine-topiramate combination (Qsymia) [95]	Long-term (over-the-counter use in the United States)	-Phentermine: Reduces appetite, sympathomimetic amines) -Topiramate: anticonvulsant or antiepileptic drug	60 mg/3 times/day	Dizzy spell condition, xerostomia symptom, sleep disorder
Orlistat: Alli, Xenical [96]	Long term	Reduces fat absorption	120 mg 3 times/day (Capsule)	Gut disorder
Bupropion-naltrexone (Contrave) [97]	Long term	Decreases appetite, cravings	1 tablet (Bupropion HCI, 90 mg: naltrexone HCI, 8 mg/day up to (360 mg:32 mg)	Bowel problem, xerostomia symptoms, sleep disorder
Liraglutide 3.0 mg (Saxenda) [98]	Long term	Decreases appetite, increases satiety	3 mg/day (Injectable)	Dizzy spell condition, abdominal pain
Gelesis100 (Plenity) [99]	Long term	Increases satiety.	1 dose of 3 oral capsules (2.25 g/dose	Gut problem, abdominal pain, bowel disorder
Setmelanotide (Imciveree) [100]	Long term	Decreases appetite.	2 mg/2 times/day or up to 3 mg (injection option)	Skin irritation at injection area, dizzy spell, sensation problem
Semaglutide 2.4 mg (Wegovy) [101]	Long term	Decreases appetite, increases satiety	0.25, 0.5, 1.7 and 2.4 mg, 1.0 g (Ozempic) Injection (optional tablet mode)	Bowel problem, skin irritation at injection area, dizzy spell
Tirzepatide [102]	Long term	Decreases appetite, increases satiety	5, 10, or 15 mg/weekly	Mild effect due to dose increases

### Drug discovery

The fundamental strategy to combat obesity includes the adoption of healthier eating patterns and engaging in consistent to moderate physical activity. However, despite initial significant weight loss, many individuals find it challenging to adhere to these lifestyle modifications, often experiencing a rebound in weight within 2 years [35].

Although pharmacological treatments have progressed, effectively integrating them with sustainable lifestyle changes remains a major hurdle in the successful management of obesity. Drug discovery can be related to the lipid pathways and molecular mechanisms due to its roles in various biological processes. Basically, lipids play crucial roles in energy storage, cell membrane structure, and signaling. By targeting specific enzymes, transport proteins, and regulatory pathways, new drugs can be developed to modulate lipid metabolism in a way that is both effective and tailored to individual patient needs [19]. The initial stage of lipid metabolism begins with the digestion of dietary fats. Pancreatic lipase plays a pivotal role in this process by hydrolyzing triglycerides (TG) into free fatty acids (FA), and monoglycerides (MG) in the small intestine [17]. While bile salts, secreted by the liver, emulsify dietary fats, enhancing the action of pancreatic lipase. They also form micelles that incorporate lipid digestion products, facilitating their transport to the intestinal mucosa for absorption [19]. The process of lipid digestion and absorption is crucial for the bioavailability of lipophilic drugs. Drugs designed to exploit these pathways, such as lipophilic prodrugs, can be incorporated into micelles for enhanced solubilization and absorption. This approach is particularly valuable for drugs that require high fat-solubility for efficacy, such as certain anti-cancer agents or drugs targeting metabolic disorders [5, 17, 19]. Furthermore, lipogenesis refers to the synthesis of fatty acids from acetyl-CoA and subsequent conversion into triglycerides, where excess glucose and other carbohydrates are converted into fatty acids for storage. This pathway is regulated by insulin, which promotes the activation of acetyl-CoA carboxylase (ACC) and fatty acid synthase (FAS), the key enzymes in fatty acid synthesis. Excessive lipogenesis can lead to the accumulation of fat in the liver (hepatic steatosis) and other tissues, contributing to obesity and metabolic syndrome. Thus, targeting the enzymes involved in lipogenesis is a promising strategy for the development of anti-obesity and anti-diabetic drugs. For example, ACC inhibitors are being explored for their potential to reduce lipogenesis and thus decrease fat accumulation in the liver and other tissues [12, 17, 19, 20].

The creation of anti-obesity drugs is crucial for those who struggle to lose weight through diet and exercise alone. Yet, the availability of these medications is limited due to safety concerns, with some being removed from the market due to adverse body health impacts.

Therefore, the quest for effective obesity treatments, especially those that target pancreatic lipase for the reduction of fat absorption, reveals both challenges and opportunities. The contemporary therapeutic landscape against obesity mainly includes three key dominant agents: orlistat, a lipase inhibitor; sibutramine, a reuptake inhibitor of serotonin and norepinephrine; and catecholamine-lipolytic regulators [16, 28, 36]. To better understand obesity, it is crucial to explore the molecular mechanisms by which pancreatic lipase (PL) influences lipid metabolism and fat accumulation. Deeper insights into PL's role could reveal novel targets for more effective obesity treatments [36].

In the context of new drug discovery, particularly for anti-obesity treatments, addressing the cost-effectiveness and accessibility of these innovations is critical for their widespread clinical adoption. Addressing the cost-effectiveness and accessibility of new anti-obesity treatments is crucial for ensuring that these innovations reach a broader population and provide equitable health benefits. While novel therapies, such as those derived from nanotechnology or personalized medicine, hold great potential for improving treatment outcomes, their high development costs and complexity can limit accessibility for patients. Cost-effectiveness is a key consideration in determining whether a new treatment can be sustainably integrated into healthcare systems. For anti-obesity drugs to be widely adopted, they must not only demonstrate clinical efficacy but also provide a favorable cost–benefit ratio compared to existing treatments. This involves ensuring that the costs associated with drug development, production, and delivery do not make the therapy prohibitively expensive for patients or healthcare providers. Lowering costs can be achieved through innovative manufacturing techniques, economies of scale, and streamlined regulatory pathways [36–38]. Other than that, the role of epigenetics in drug reaction is being unveiled through extensive analyses and innovative data analysis, underscoring the significance of incorporating epigenetic insights into pharmacological planning [38]. Even with advanced pharmacological treatments, the integration of these therapies with effective lifestyle changes continues to be a complex and ongoing challenge. The preference for high-content phenotypic screening in the drug discovery process reflects its comprehensive capacity to generate multi-dimensional data specific to each drug, thereby refining the approach to identifying new therapeutic agents [36, 37].

As Orlistat is one of the drugs that inhibit pancreatic lipase, it has been subjected to extensive research due to its ability to aid in weight loss, yet its potential side effects have been revealed. Mechanistically, by covalently attaching to the serine residue in the pancreatic lipase's active site, orlistat deactivates the enzyme. This prevents the breakdown of dietary fats into free fatty acids and monoglycerides that can be absorbed, leading to a reduction in caloric intake and weight loss [24]. Studies indicate that orlistat not only contributes to significant weight reduction but also ameliorates cardiovascular risk factors in overweight individuals [39]. Despite its effectiveness, the drug's gastrointestinal side effects due to its action on gastrointestinal lipases can affect patient compliance [40]. Orlistat works by stopping the digestion and absorption of fats from the diet, which supports weight loss management [41]. Beyond its role in weight loss, orlistat has been shown to positively influence the levels of adiponectin, leptin, and C-reactive protein in the plasma, hinting at its wider metabolic benefits. Investigations into orlistat have also highlighted its potential in exerting anti-tumor effects, showcasing its pharmacological versatility. Current PL inhibitors like orlistat are constrained by limited long-term effectiveness and safety, highlighting the necessity for developing more effective and safer options for managing obesity [32, 41].

Comparative studies on the safety and efficacy of orlistat against other weight-loss drugs suggest that the side effects linked to orlistat are generally minor and temporary [42]. Its distinct action of inhibiting fat absorption in the intestines could also play a role in lowering levels of circulating free fatty acids and improving insulin sensitivity [43]. Nonetheless, orlistat is associated with an increased rate of gastrointestinal adverse reactions and a noticeable reduction in the uptake of fat-soluble vitamins.

Orlistat has demonstrated effectiveness in managing weight and diminishing the accumulation of harmful visceral fat, yet some users experience adverse reactions and issues with tolerability [33]. Research also explores its potential in hindering the growth of cancer cells and cardiovascular health improvement, suggesting wider therapeutic uses [40]. Nonetheless, the consideration of its potential gastrointestinal side effects and issues with tolerability is important in clinical decisions. Addressing and reducing side effects is a pivotal aspect of medical treatments and the advancement of new pharmaceuticals [41, 42]. Gradual drug titration can mitigate central nervous system side effects by allowing the body to develop pharmacodynamic tolerance [44]. Additionally, analyzing drug side effects offers insights into new drug–target interactions, providing opportunities for drug re-purposing [45]. Employing a holistic approach to drug development can help in reducing potential drug side effects. Tailoring dosing schedules is crucial for the optimal use of antibiotics and in reducing adverse drug reactions [45]. Furthermore, there is a push towards creating drug delivery systems that enhance drug bioavailability and reduce undesirable effects.

The FDA has approved six drugs for the long-term treatment of obesity, Contrave (bupropion and naltrexone), Saxenda (liraglutide), Xenical and Alli (orlistat), Qsymia (phentermine and topiramate), Wegovy (semaglutide), and Imcivree (setmelanotide) [43, 45]. These medications mainly work by reducing hunger or enhancing satiety, with the exception of orlistat, which blocks the body's fat absorption [46]. Contrave, blending addiction and depression therapies, may elevate the risk of suicide and blood pressure, and commonly causes nausea and headaches [46]. Saxenda, an injectable for diabetes management, frequently leads to nausea and vomiting. Qsymia combines a weight-loss stimulant with an epilepsy drug, carrying risks of abuse, heightened heart rate, and possible birth defects. Administered weekly, semaglutide can cause gastrointestinal issues and fatigue [47]. Approved for those over six with specific genetic conditions, Imcivree can decrease hunger and increase resting calorie expenditure, with possible side effects including skin issues, nausea, and changes in mood. Additionally, aminorex and sibutramine have been linked to cardiovascular toxicity, pulmonary hypertension, and a rise in non-fatal cardiovascular incidents [37, 39, 43, 45, 46]. These medications necessitate careful selection and monitoring by healthcare providers due to the potential for side effects and specific application criteria, such as genetic testing for eligibility.

Moreover, this research investigates how astaxanthin, along with other xanthophylls like zeaxanthin and violaxanthin derived from plant and marine foods, can block pancreatic lipase, an essential enzyme in fat digestion. Initial in-silico studies showed these xanthophylls had a higher potential for inhibiting the enzyme compared to orlistat, a drug known for its lipid-lowering effects. However, when these findings were tested under conditions that simulate the natural process of lipid digestion in the gastrointestinal tract, using a method known as the potential of Hydrogen (pH)-stat test, the expected inhibitory effects were not observed. Particularly, sea buckthorn oil, which is rich in native xanthophylls, actually showed an increase in lipolysis by about 22% compared to oils with lower xanthophyll content. This suggests that while xanthophylls can inhibit pancreatic lipase in theoretical models, their amphiphilic nature might enhance the formation of emulsions during actual digestion, thereby increasing the breakdown and absorption of fats rather than inhibiting it [34].

### Natural product

Recent studies have highlighted the potential of natural substances in offering alternative treatments for obesity by focusing on their capacity to inhibit pancreatic lipase among other pathways. These natural remedies contribute to weight management through various actions, including boosting metabolism, controlling blood sugar levels, reducing appetite, hindering essential digestive enzymes, enhancing insulin sensitivity, limiting new fat cell formation, and promoting the breakdown of existing fat cells [48]. Key proteins and molecular pathways, such as uncoupling protein-1 and AMP-activated protein kinase, are crucial in the transformation of white adipose tissue into brown adipose tissue, facilitating fat breakdown. Additionally, certain natural substances are known for their ability to reduce inflammation by lowering pro-inflammatory markers and modulating adipokines, which are crucial for managing body weight.

Specifically, plant-derived phytochemicals have been recognized for their effectiveness in combating obesity by inhibiting digestive enzymes like pancreatic lipase and amylase, suppressing appetite, limiting the development of white adipose tissue, or encouraging its conversion to brown adipose tissue. These substances also stimulate proteins that play a role in fat metabolism, reduce levels of the hunger hormone ghrelin, and enhance lipid profiles in the blood. Notable examples of these phytochemicals include curcumin from turmeric, anthocyanins from blueberries, and epigallocatechin gallate from green tea. These compounds are celebrated for their capabilities in hindering the growth of fat cells, promoting the breakdown of fats, and reducing the formation of new fat cells, offering promising avenues for addressing metabolic issues [28, 48].

Previous research also delves into herbal solutions derived from plants, discussing how obesity stems from an imbalance between fat breakdown and creation, alongside poor regulation of fat cell formation. This imbalance leads to an increase in fat mass due to the expansion and transformation of adipose-derived stem cells, changes in cell shape, and gene activity [29]. Adipogenic regulators such as PPARG and the CEBP family are key in this process, stimulating the expression of proteins essential for fat cell maturation. Additionally, during fat tissue expansion, these stem cells play a significant role in promoting new blood vessel formation and increasing fat content within cells. Herbal compounds like celastrol, extracted from Tripterygium wilfordi, have been identified for their potential in obesity treatment by enhancing leptin sensitivity and inhibiting new blood vessel growth, which may contribute to their effectiveness in reducing fat mass [32]. Celastrol specifically targets the later stages of fat cell development, decreasing fat accumulation by downregulating genes involved in fat cell formation, thus highlighting its potential as a pharmacological strategy in obesity management [26, 49].

Overall, these findings advocate for further exploration of natural product's potential in the innovative treatment strategy for obesity management, and leveraging their diverse mechanisms of action. Their role is to inhibit the development of fat cells, encouraging the breakdown of fats, and reducing the synthesis of new fat cells, thereby helping to manage obesity and related metabolic conditions [26, 28].

In addition, a parallel study has discussed the alternative to combating obesity, which is related to herbs derived from plants [50]. They discussed, in general, the results of obesity related to from the excessive growth and multiplication of fat cells (adipocytes) due to a disrupted balance between fat breakdown (lipolysis) and fat creation (lipogenesis), alongside poor regulation of fat cell formation (adipogenesis). This process is marked by an increase in fat mass, driven by the growth and transformation of adipose-derived stem cells (ADSCs), alongside changes in cellular morphology and genetic activity that contribute to the enlargement of adipose tissue. Central to this process are adipogenic regulators, such as peroxisome proliferator-activated receptor gamma (PPARG) and the CCAAT/enhancer-binding protein (CEBP) family. These regulators play a pivotal role in initiating the expression of vital proteins necessary for the maturation of fat cells, including those involved in lipid binding, synthesis, and processing. Additionally, as adipose tissue expands, ADSCs play a critical role in fostering new blood vessel formation, boosting the adipogenic potential of stem cells within the tissue, and increasing the lipid content within adipocytes. An emerging approach involves leveraging renowned natural compounds from herbal medicine, such as celastrol, which is celebrated in traditional Chinese medicine for its therapeutic potential in various conditions, including obesity. Celastrol enhances leptin sensitivity, an essential hormone for regulating appetite and metabolism [32]. Research highlights celastrol's capability to suppress angiogenesis, shedding light on its efficacy in diminishing fat mass. Particularly, the application of celastrol during advanced stages of adipocyte differentiation has been observed to curtail fat storage in human adipose-derived stem cells (hADSCs) by suppressing critical genes implicated in adipogenesis [50].

These findings indicate celastrol's potential to decrease fat storage and impede the maturation of fat cells by inhibiting the action of PPARG, CEBPA, and fatty acid-binding protein 4 (FABP4). The work underscores celastrol's promise as a pharmacological option for combating obesity and its metabolic repercussions, advocating for further investigation into its mechanisms and therapeutic applications [50].

The review suggests that exploring natural products more thoroughly could lead to more effective and safer obesity treatments by harnessing these diverse mechanisms [28, 50].

Recent studies have highly investigated alternatives to current anti-obesity treatments, targeting natural inhibitors of pancreatic lipase, a key enzyme in the digestion of dietary fats. Specifically, a mixed extract from *Diospyros kaki* fruit and *Citrus unshiu* peel (PCM) is found to be a potential natural inhibitor that could aid in obesity prevention and treatment [6].

At its core, investigating the role of pancreatic lipase in addressing obesity is a dynamic and hopeful field of inquiry. By probing into the intricacies of inhibiting pancreatic lipase, assessing available treatments, confronting existing hurdles, and embarking on new explorations, this research domain endeavors to furnish a thorough insight into both the present and potential future of pancreatic lipase inhibition as a strategy for obesity treatment. This endeavor is dedicated to fostering the creation of innovative and more effective therapeutic options for tackling the widespread challenge of obesity [51].

In this context, examining pancreatic lipase's contribution to obesity management unveils a fertile ground for innovation in therapeutic methods. Concentrating on the intricacies of pancreatic lipase (PL) inhibition, scrutinizing prevailing treatments, surmounting current obstacles, and pioneering future breakthroughs, this discussion aims to deliver an all-encompassing view of the role of PL inhibition in the fight against obesity. It charts a course toward fresh discoveries and progress in addressing this prevalent health dilemma.

### New finding as alternative obesity treatment: PL inhibitory peptides approaches

The previous study explores the potential of natural peptides as inhibitors of pancreatic lipase, aiming to contribute to obesity treatment. Basically, peptides represent a distinct category of biochemical entities, positioned between small molecules and proteins in terms of molecular size, yet they differ significantly in their biochemical and therapeutic properties. Serving as essential signaling agents across numerous bodily functions, peptides hold considerable promise for medical treatments due to their ability to naturally emulate biological processes. Their rapid breakdown in the human body also makes them a relatively safe option for use. Recently, scientists have found 176 pancreatic lipase inhibitory peptides from various sources, including plants-based sources (millet grain, rice bran [52], and cumin seeds), marine life such as those extracted from sea cucumbers, tuna, shark, and dried spirulina seaweed [53]. The peptides-derived insect sources such as tropical house crickets, mealworms, desert locusts, and dairy sources, including camel and cow casein [54]. These peptides, identified through bioinformatic analyses and molecular docking, show promise due to their structural activity relationships and interaction mechanisms. Unlike weight loss medicine, weight loss devices, bariatric surgery, and liposuction, which may have significant risks or side effects, traditional weight loss methods, such as naturally derived peptides offer a safer, more accessible alternative. The investigation underlines the peptides' hydrophobic nature and their role in reducing macronutrient absorption, highlighting their potential as natural, effective anti-obesity agents [52–54].

In short, Obesity, now recognized as a significant medical concern on the current anti-obesity medications works by suppressing appetite through the elevation of neurotransmitters, inhibiting digestive enzymes, and slowing down gastric emptying. However, their side effects on the cardiovascular, psychological, and gastrointestinal systems have prompted the search for safer treatments. Phytochemicals from plants offer a promising alternative, displaying similar beneficial mechanisms with fewer side effects. Notably, natural compounds uniquely contribute to weight management by promoting the browning of white adipose tissue, enhancing thermogenesis, encouraging fat cell death, and blocking fat cell formation [28]. These effects are mediated through several mechanisms, including metabolic stimulation, appetite regulation, inhibition of digestive enzymes, improving in insulin sensitivity, and direct action on fat storage and breakdown processes. Critical proteins and molecular pathways like UCP-1, PRDM16, PPAR-γ, SIRT1, and AMPK play roles in these processes, influenced by phytochemicals to effectively combat obesity through multifaceted actions on fat metabolism and energy expenditure [28, 55].

### Advance emergence-related obesity treatments

In the context of emergence and advanced treatments, peptides, which are a short chain of amino acids are leveraged as precision-targeting agents because of their specificity in binding to designated receptors or biomarkers. These peptides, whether naturally occurring or synthetically crafted, serve dual roles: they are used either as direct drug conjugates or as delivery transporters for drugs encapsulated in nanoparticles. This approach holds significant potential for the treatment of a variety of diseases, including cancer, cardiovascular diseases, and neurological conditions [35, 36]. Beyond peptides, targeting molecules like aptamers (short DNA or RNA strands that bind specifically to targets), small molecules (designed to interact with disease-related receptors or enzymes), and carbohydrates (for targeting specific cell surface lectins or receptors) are also under investigation for their therapeutic potential [56–60].

The advent of nanotechnology has paved the way for the development of sophisticated targeted drug delivery systems (DDSs) that leverage nanoparticles engineered with functional capabilities [61]. Emerging technologies like nanotechnology hold great promise for revolutionizing anti-obesity treatments by enabling more precise drug delivery and boosting therapeutic effectiveness. Nanomedicine offers a promising approach to address this gap by enabling more precise and effective anti-obesity therapies. Nanoparticles can be engineered to deliver drugs directly to adipose tissue, enhancing the efficacy of weight-loss agents while minimizing systemic side effects. Additionally, nanocarriers can be designed to improve the bioavailability of poorly soluble drugs, allowing for lower dosages and reducing the risk of adverse reactions. These advanced nanocarriers are crafted to carry both targeting molecules and therapeutic compounds directly to the afflicted cells or tissues. This is achieved through surface modifications with targeting ligands, such as antibodies or peptides, enhancing their ability to be taken up by cells and localized precisely at the site of the disease. Despite its potential, the integration of nanomedicine into clinical practice faces challenges, including the need for rigorous safety evaluations, immune reactions, regulatory approval, long-term stability, and the development of scalable manufacturing processes alongside ethical frameworks. Nonetheless, the targeted nature of nanotechnology in drug delivery could revolutionize obesity treatment by providing personalized crowd support with an integrative-care model and more effective interventions. Personalized medicine holds significant potential for advancing anti-obesity treatments by tailoring therapies to individual genetic profiles and metabolic responses, thereby enhancing treatment efficacy and minimizing adverse effects.

In the realm of creating targeted DDSs, there are three prominent synthetic methods for producing polymer–drug conjugates: binding therapeutic compounds to synthetic polymeric carriers, incorporating therapeutic molecules with monomers prior to the polymerization process, and embedding drugs into polymers during their formation [62]. These innovative conjugates bring forth numerous benefits, such as improved drug bioavailability and biodegradability, minimized drug toxicity, increased water solubility and stability, enhanced biocompatibility, and precise control over drug release. These advantages are crucial for overcoming drug resistance. Nonetheless, deploying polymer–drug conjugates, particularly in multi-drug regimens, presents hurdles, including identifying the ideal drug-to-therapeutic agent ratios and addressing concerns related to low drug loading capacities [56, 57, 60].

Furthermore, the advanced emergence has been explored in RNA-targeted drug discovery and RNA bioengineering is providing new insights into the dynamic structure landscape of RNA, which has implications for drug discovery [63]. Even obesity is a complex issue influenced by multiple factors. Key to understanding these conditions is the role of adipocytes (fat cells) and the process of adipogenesis (the formation of fat cells). Central to the development of obesity is the growth of white adipose tissue (WAT), marked by both an enlargement (hypertrophy) and a proliferation (hyperplasia) of fat cells, or adipocytes. WAT is composed not only of adipocytes but also mesenchymal stem cells (MSCs) which serve as precursors to these fat cells, in addition to containing endothelial and immune cells [30]. MSCs have the potential to evolve into new adipocytes, which subsequently store triglycerides within their lipid droplets [50, 63]. By delving into the dynamics of adipose stem cells and the adipogenesis process, scientists can uncover innovative avenues for tackling obesity. The complexity of adipogenesis, involving numerous molecular mechanisms, makes the study of regulators of this process and their potential as anti-obesity medication targets (AOM) particularly valuable. This approach includes exploring RNA functions, gene expression, and metabolic regulation during the adipogenesis of human mesenchymal stem cells (MSCs) and the breakdown of fats in lipolysis [50, 63]. The exploration of treatments for obesity has advanced into areas of RNA and stem cell research, focusing on the control of fat cell formation (adipogenesis) in mesenchymal stem cells (MSCs). This is seen as a promising approach for addressing people with obesity and related conditions associated with aging.

## Orlistat: Understanding the pharmacokinetic efficacy and potential side effects

Orlistat is a well-researched pancreatic lipase inhibitor recognized for its weight reduction capabilities and positive impact on cardiovascular risk markers in patients with obesity. Despite its effectiveness, the drug's gastrointestinal side effects, stemming from its action of blocking fat absorption, can affect adherence. Orlistat works by hindering the absorption of dietary fats, leading to weight loss, and also shows potential in enhancing metabolic markers like adiponectin and leptin levels [32, 33]. Beyond its weight loss benefits, scientists have suggested that orlistat might have anti-tumor [64].

In the context of understanding the pharmacokinetic efficacy of Orlistat, along with its potential side effects, can be influenced by obesity through changes in drug absorption, metabolism, distribution, and excretion. The way a drug is administered plays a crucial role in its systemic availability, with routes like intravenous, subcutaneous, and intramuscular bypassing the liver initially, thus affecting systemic bioavailability. Numerous pharmaceuticals are formulated as ionized salts, incorporating elements like sodium or potassium, to enhance their pharmacokinetic profiles. This includes facilitating dissolution in the gastrointestinal tract to improve absorption, extending the product's shelf life, and streamlining the manufacturing process. Medications such as phentermine hydrochloride (HCl), naltrexone HCl, and bupropion HCl are examples where the salt form significantly influences their pharmacokinetic behaviors.

When compared to other medications for obesity management, orlistat stands out primarily due to its gastrointestinal side effects which are typically manageable. Its distinctive mechanism of action reduces the absorption of fats, potentially benefiting insulin sensitivity and reducing levels of circulating fatty acids. However, concerns about its impact on gastrointestinal health and the potential for decreased absorption of fat-soluble vitamins persist. Despite these challenges, orlistat is acknowledged for its effectiveness in weight management and its potential for anti-cancer research, mainly attributed to its role as a gastrointestinal lipase inhibitor that limits dietary fat absorption [39, 41, 42].

Thus, orlistat is effective in weight management and poses cardiovascular benefits, yet its gastrointestinal side effects and impact on vitamin absorption necessitate careful consideration. Ongoing research into its various pharmacological effects and potential beyond obesity treatment is encouraged.

### Adverse effects: addresses current clinical challenges with Orlistat-related gastrointestinal

Orlistat, aimed at treating obesity, effectively aids in weight loss but is frequently linked to adverse gastrointestinal disorder reactions including oily stools, diarrhea, stomach pain, and fecal incontinence. The gastrointestinal side effects associated with certain treatments play a crucial role in clinical practice due to their impact on patient adherence to therapy. Orlistat operates by inhibiting enzymes in the gastrointestinal tract responsible for fat digestion, consequently reducing fat absorption by ~30% [65]. Additionally, challenges in its formulation due to poor solubility and low oral bioavailability complicate its clinical use (Table 1).

Scientists focusing on the specific adverse effects of fecal spotting and other related issues underscores the necessity for a deeper comprehension of these reactions and the development of management strategies to mitigate them. Studies have also delved into orlistat's impact on lipid metabolism and its potential link to cancer, finding no substantial influence on the progression of colorectal cancer [43]. Moreover, its utility alongside lifestyle modifications in treating conditions like polycystic ovary syndrome highlights its potential advantages for certain groups.

In essence, while orlistat's role in weight reduction is established, its associated gastrointestinal adverse effects present significant hurdles in clinical practice. There is a pressing need for further investigation to understand these effects better and to devise effective management approaches that could enhance patient experience and adherence to orlistat treatment.

### Adverse effects: Orlistat-associated to liver failure

Orlistat, a drug inhibiting pancreatic lipase and aimed at aiding weight loss, has been linked to severe liver-related side effects, including the formation of gallstones, liver inflammation with bile obstruction, and acute liver failure, as highlighted in various studies [5, 42, 66–69]. Research findings have underscored instances of grave liver conditions connected to the consumption of orlistat, leading to regulatory in safety guidelines regarding its use [5, 68]. In addition, orlistat's potential to cause liver damage and its association with severe liver outcomes, such as gallstone formation, obstructive jaundice, and acute liver failure, have been documented [68, 70]. The drug has also been related to the formation of silent gallstones and significant weight reduction in certain individuals [42]. Despite the low incidence of serious side effects and discontinuation of treatment due to these effects reported in clinical trials, the prevalence of gallstones and obstructive jaundice linked to orlistat demands attention [42, 71]. It is crucial to acknowledge that the occurrence of adverse events, especially those affecting the liver due to orlistat, might be underrepresented in published reports [72]. Therefore, the potential for serious hepatic adverse effects should be carefully considered when prescribing orlistat for weight management.

### Adverse effects: Orlistat-associated acute kidney injury and enteric hyperoxaluria

Investigating the reflection of Orlistat, acute kidney injury, and enteric hyperoxaluria is vital due to the rare yet serious adverse effects linked to Orlistat. Enteric hyperoxaluria, which results from enhanced oxalate absorption in the intestine, is known to cause kidney stones, chronic kidney damage, and acute oxalate nephropathy [73, 74]. The mechanisms involved encompass numerous gastrointestinal disorders that lead to poor fat absorption and reduced secretion of oxalate in the gut, which are linked to hyperoxaluria associated with obesity [75]. Moreover, secondary hyperoxaluria, prompted by enhanced absorption of dietary oxalate, can increase the risk of developing kidney stones and oxalate nephropathy, especially in individuals with gastrointestinal complications or those who have undergone intestinal surgeries [76]. This section underscores the importance of investigating the relationship between enteric hyperoxaluria and the onset of acute kidney injury, particularly in the context of conditions such as inflammatory bowel disease, short bowel syndrome, and following bariatric surgery [75].

## Pharmacodynamics of orlistat on drug absorption and interaction complications

Orlistat, known for its effectiveness in weight management by blocking lipase enzymes, significantly affects the absorption and effectiveness of various medications, including warfarin, amiodarone, ciclosporin, and thyroxine, as well as fat-soluble vitamins [77]. This interference is primarily due to orlistat's ability to reduce fat absorption, which in turn impacts the bioavailability of these medications. Key interactions, such as reduced cyclosporin absorption leading to Evan's syndrome relapse, and enhanced effects of warfarin's anticoagulation due to decreased vitamin absorption, underline the importance of vigilant monitoring and potentially supplementing vitamins in individuals taking orlistat [39, 42].

These observations, derived from various studies and case reports, emphasize the critical need for healthcare providers to be vigilant about potential drug interactions when prescribing orlistat, especially for patients on concurrent medications that might be impacted by altered lipid absorption dynamics. Orlistat operates by inhibiting your body's ability to absorb some of the fat from the foods you consume. It is advised to take it with water either during your meal or within an hour afterward [43]. Given orlistat's potential to impair the absorption of certain vitamins, incorporating a daily multivitamin supplement is recommended, which should be consumed either two hours before or after taking orlistat, or at bedtime. To reduce the likelihood of side effects, it is important to ensure your diet does not exceed 30% fat and maintains a balanced distribution of fats, carbohydrates, and proteins across all three meals. Following a low-calorie diet and exercise regimen, as advised by your healthcare provider is essential. For individuals on cyclosporine or levothyroxine, it is important to time these medications to avoid interference with orlistat; take cyclosporine at least three hours before or after orlistat, and levothyroxine four hours apart from orlistat [43, 78].

## Evolution and challenges of new obesity drugs related to adverse effects and future direction

Combatting obesity represents a major health dilemma, as it contributes to a heightened risk of disease, diminishes the quality of life, and can reduce life expectancy. Despite initiatives aimed at prevention and treatment on both personal and societal fronts, achieving success is challenging. Attempts to implement lifestyle modifications, such as lowering calorie consumption and enhancing physical activity, are often frustrated by complicated adaptations involving hormones, metabolism, and brain chemistry that oppose weight loss and promote the rebound of weight [31, 43]. Although weight-loss surgery shows effectiveness in severe cases, complications like weight regain post-surgery remain a concern. Less invasive methods like intra-gastric balloons offer a temporary solution but cannot rival the outcomes of bariatric surgery. Pharmacological interventions have advanced, yet many anti-obesity drugs fail in clinical trials due to severe side effects, limited usage durations, and moderate long-term efficacy. Some new studies on new therapeutic opportunities and drug delivery have been investigated (Table 2). Studies have shown the beneficial effects of long-term use, particularly with natural molecules, as they often present fewer side effects and improved safety profiles. Recent research on new therapeutic opportunities and drug delivery methods highlights the potential of these natural compounds in providing sustainable anti-obesity solutions [79].

**Table 2:** Overview of new therapeutic opportunity and drug delivery in anti-obesity medication information.

Compound	Source	Principle	Delivery mode	Study type
*C. fimbriata*	*Caralluma fimbriata* extract	Downregulate ghrelin production & neuropeptide (NPY)	1 g/day orally	Clinical trial [103]
6-gingerol	Ginger extract	Decrease PPARy, C/EBPa & FABP4 expression, increase adiponectin expression	25 mg/kg/day	In vivo [104]
Protocatechuic acid	Cinnamon extract	Browning effect, increased expression UCP1 and brown adipocytes marker	80 µg/ml/day	In vitro trial [105]
Flavonoids, glycosides, phenolic acids, and tannins	*Alchemilla. monticola* extract	Suppresses the adipogenic markers PPARγ, C/EBPα, and adiponectin	5, 10 and 25 μg/ml/day	In vitro trial [106]
Delphinidin, Anthocyanins	Vegetables, fruits, flowers, leaves, and seeds	Suppressing the adipogenesis markers increases the fatty acid metabolism gene in 3T3-L1	25, 50 and 100 µM of delphinidin-3-O-β-glucoside	In vitro trial [107]
Ginsenoside	Black ginseng (BG)	Triggered browning effects in 3T3-L1 and primary white adipocytes (PWATs)		In vitro trial [55]
Dibenzazepine-loaded nanoparticles	Synthesis based	Local browning of white adipose tissue	Injected DBZ-NPs into the inguinal WAT depots (1 mg/mL Cy5.5-conjugated PLGA NPs)	In vivo & in vitro trials [85]
Amino acid sequence for this peak, identified as FYLGYCDY (peptide approach)	De-oiled rice bran (DORB) (by-product of the rice bran oil industry)	DORB protein hydrolysate has lipase inhibitor activity.	2.5 g of DORB powder in 50 mL of 20 mM phosphate buffer at a pH of 8.0.	In vitro [52]

Recently, scientists have identified the advanced technology and key pathways, such as nanotechnology drug carriers involving RNA molecular or other mechanisms, such as the leptin-melanocortin and GLP-1/GLP-1R systems that are crucial in energy balance and appetite regulation [80, 81]. GLP-1R agonists show promise in weight management however, challenges remain in enhancing their stability and effectiveness in the clinical setting due to natural degradation processes [79, 80].

GLP-1, a peptide hormone composed of 30 or 31 amino acids, is synthesized in the intestines and plays a pivotal role in boosting insulin secretion while lowering glucagon levels under normal physiological conditions. It was identified in 1983 as a product of proglucagon conversion by L cells in the lining of the intestines and is also produced by pancreatic α-cells, as well as being present in islet β-cells and the intestine [82]. Crucial for the regulation of glucose tolerance, GLP-1 acts through GLP-1 receptors, which belong to the glucagon receptor family and are located in several tissues, including the pancreas and heart [79]. GLP-1 receptor agonists, targeting the entero-insular axis, mimic the insulin-boosting effects of bariatric surgery. These drugs offer a novel approach to obesity treatment, although their precise action mechanism and long-term safety and effectiveness require further clinical validation [83].

The exploration of nanotechnology-based therapies has unveiled potential solutions to overcome the drawbacks of conventional anti-obesity medications, including their nonspecific actions and the temporary impact on weight reduction. Emerging nanotechnology-based therapies should be thoroughly evaluated for their potential in anti-obesity treatment, as they offer innovative approaches for targeted drug delivery and improved therapeutic outcomes. A comprehensive examination of these technologies could lead to significant advancements in managing obesity [84]. The pressing need for novel interventions has directed scientific research towards nanotechnology as a viable route for crafting more efficient treatments for obesity. A comprehensive review identifies three innovative nanotechnology-driven strategies aimed at targeting and altering white adipose tissues (WATs) and their vasculature to combat obesity. These methods encompass obstructing the vascular supply to WATs, transforming WATs into thermogenic brown adipose tissues (BATs), and utilizing photothermal techniques to dissolve WATs [85]. Utilizing a range of nanocarriers, including liposomes, polymeric nanoparticles, and gold nanoparticles, these nanotechnology strategies promise enhanced efficacy, greater tolerance, and fewer adverse effects compared to traditional treatment modalities. This progress indicates that targeted nanotherapies may offer a revolutionary approach to addressing obesity and its related health complications.

In parallel with new drug studies, myriad scientist actively works on that. However, when employing strategy-related pharmacotherapy for obesity management, scientists and healthcare providers should adhere to several core guidelines [86]. Firstly, understanding any contraindications and side effects contributed to extensive use. Recognizing obesity as a chronic condition, there is a necessity for continuous use of anti-obesity medications (AOMs) as part of an all-encompassing treatment plan, integrating dietary, exercise, and psychological strategies. Stopping medication often results in weight gain. Secondly, AOMs are designed to target specific biological pathways influencing weight. Thirdly, the objective of treating obesity extends beyond weight loss to include the prevention of obesity-related complications and improvements in comorbid conditions, as evidenced by positive changes in cardiometabolic risk factors observed in Phase 3 clinical trials [87]. Employing a combination of AOMs that work through different mechanisms can effectively address the multifaceted nature of obesity and counteract metabolic adjustments that hinder weight loss. For instance, a patient who has hit a weight plateau with metformin might benefit from the addition of an appetite suppressant like phentermine or the combination of phentermine/topiramate to counteract the increase in hunger often caused by elevated ghrelin levels following significant weight loss through dietary changes. Lastly, there is variability in individual responses to AOMs, although when paired with lifestyle changes, these medications generally lead to significantly more weight loss compared to placebo. Thus, they are supported by stringent Federal Drug Administration (FDA) guidelines ensuring their efficacy and safety [87].

The future direction of obesity treatment is poised for significant advancements with medical professionals, policymakers, and the pharmaceutical industry recognizing the urgent need for effective and safe weight-loss medications. A new wave of anti-obesity medications is making strides in treatment options, heralding a more tailored approach to managing the condition. Currently, a range of anti-obesity medications (AOMs) are in development, showing encouraging outcomes. Noteworthy innovations include dual-action gastrointestinal peptide modulators like tirzepatide and cotadutide, which are demonstrating promise in preliminary trials. In addition, setmelanotide is pioneering a personalized medicine approach to obesity, specifically targeting very rare genetic disorders such as pro-opiomelanocortin (POMC) deficiency, proprotein convertase subtilisin/kexin type 1 (PCSK1) deficiency, and leptin receptor deficiency. Personalized medicine holds significant promise in tailoring healthcare to individual genetic, environmental, and lifestyle factors, thereby enhancing treatment efficacy and reducing adverse effects. By leveraging advancements in genomics and molecular diagnostics, personalized approaches can identify unique patient profiles, allowing for the selection of targeted therapies that align with the specific biological mechanisms driving disease in each patient [88]. This evolution in medicine also presents challenges, such as the need for robust data integration, ethical considerations, and the development of cost-effective strategies that can be widely implemented in clinical practice. In the context of personalized medicine, anti-obesity drugs play a crucial role in targeting and inhibiting the specific pathways involved in the pathogenesis of obesity. Tailoring these treatments to individual patient profiles can enhance their efficacy in preventing disease progression. [89].

On another front, semaglutide 2.4 mg is in the process of seeking approval in both the United States and the European Union, offering significant potential for individuals with obesity or overweight and related comorbidities, as evidenced by its substantial efficacy in phase 3 trials [88]. The landscape of diabetes treatment is also witnessing advancements with the introduction of semaglutide, a GLP-1 receptor analog. Tirzepatide, which combines the effects of glucose-dependent insulinotropic polypeptide and GLP-1, is undergoing phase 3 research for obesity treatment. Meanwhile, bimagrumab is exploring a new mechanism in phase 2 studies, sparking significant interest in its potential to transform the pharmacotherapy of obesity [90–92].

## Conclusion

In summary, focusing on pancreatic lipase as a method for treating obesity presents a promising path for crafting impactful therapeutic options. Present understanding suggests that blocking pancreatic lipase activity substantially decreases fat absorption, thus supporting weight control efforts. The weight management focus on weight loss aims to enhance overall health by diminishing the harmful effects of excessive adipose tissue, while also promoting overall bodily health. However, the journey from understanding the basic mechanisms to the application of these insights in clinical settings involves overcoming several challenges, including optimizing drug efficacy, minimizing side effects, and ensuring long-term safety and tolerability. Ideally, society would foster healthy eating and active lifestyles as normative behaviors, free from the pressures of emotional and financial stress. However, recognizing the current challenges, there is a pressing need to develop more effective pharmacological solutions for those requiring assistance in weight management as a means to improve their health. Future directions should focus on advancing our understanding of the molecular interactions and regulatory pathways involved in lipid metabolism and pancreatic lipase function. Additionally, exploring the potential of combination therapies, personalized medicine approaches, and novel delivery systems could enhance the effectiveness and acceptability of pancreatic lipase inhibitors. Ultimately, interdisciplinary collaboration among researchers, clinicians, and pharmaceutical industries is crucial for translating these insights into safe, effective, and accessible obesity treatments. Furthermore, the advent of new-generation anti-obesity drugs offers promising avenues for achieving substantial and healthful weight reduction in many individuals facing obesity. This shift not only propels forward the potential for significant advancements in obesity treatment but also allows a refocus on addressing obesity directly as the underlying cause of numerous comorbidities, rather than solely managing its associated health conditions. The anticipation is that ongoing research and development in this field will equip biomedical scientists and clinical practitioners with innovative strategies and insights for combating obesity more effectively, marking a significant stride towards a holistic approach to obesity medicine.
